# Spread of awareness of COVID-19 between December 2019 and March 2020 in France

**DOI:** 10.1038/s41598-024-56423-5

**Published:** 2024-03-21

**Authors:** Horace Blanc, Eliane Rothier Bautzer, Natacha Vellut, Viet-Thi Tran

**Affiliations:** 1 Inserm, INRAE, Center for Research in Epidemiology and StatisticS (CRESS), Université Paris Cité and Université Sorbonne Paris Nord, 75004 Paris, France; 2 CNRS, INSERM, Centre for Research on Medicine, Science, Health, Mental Health, and Society (Cermes3), Université Paris Cité, 75004 Paris, France; 3 Centre d’Epidémiologie Clinique, Hôpital Hôtel-Dieu, Assistance Publique Hôpitaux de Paris, 75004 Paris, France

**Keywords:** COVID-19, Awareness, Sociology, Viral infection, Human behaviour

## Abstract

During the early phase of outbreaks, awareness of the presence of the disease plays an important role in transmission dynamics. To investigate the processes of how people become aware of a disease, we conducted two complementary investigations. First, we surveyed 868 academic researchers in France, on the time and circumstances when they became aware of COVID-19 as an important event. We found that 25% did so before February 18th (first death in France), 50% did so before March 10th (first presidential allocution) and 75% did so before March 16th (announcement of the lockdown). Awareness came from nine categories of circumstances: (1) decisions taken by the government (elicited by 35.7% participants); (2) information from media or social media (24.7%); (3) conversation with friends (22.4%); (4) observed changes in their personal lives (14.0%); (5) decisions taken by the employer (9.2%); (6) observed changes at work (9.9%); (7) suspected case of COVID-19 in their entourage (3.1%); (8) fear for oneself or their entourage (2.8%) and (9) self-appraisal of scientific reports (2.8%). Second, we appraised three general media in France (a television news show, a radio news show, and a newspaper) and showed that COVID-19 became a preeminent topic only after March 1st 2020 when the epidemic is present on national soil. Our results show that multiple intricated factors prompt the awareness of an emerging infectious disease. Awareness is not solely driven by general media as they begin to focus on the topic late.

## Introduction

In December 2019, SARS-CoV2, the pathogen responsible for Coronavirus Disease 19 (COVID-19), emerged in China. In less than 3 months, the virus had spread to over 100 countries, prompting the World Health Organization to declare a global pandemic^1^. We aim here to describe the precise time and circumstances during which individuals became aware of COVID-19 as an important event in their lives, during the very early phase of the outbreak (December 2019 to March 2020).

During outbreaks, accurate anticipation of transmission dynamics is essential to tailor and measure the effect of strategies to control the emerging infectious disease^2–4^. Among variables that affect transmission, human reactions to the presence of disease are often ignored despite playing an important role in the implementation of protective behaviours such as social distancing, wearing masks or vaccine acceptance^5–7^. In particular, models have shown that the early spread of awareness of the presence of a disease can reduce the size of an epidemic outbreak.

Awareness of an emerging infectious disease is a subjective construct, dependant from individuals’ background and personality, and prompted by individuals’ reactions to information, lived experiences or observed changes in life due to decisions from governing authorities^8^. In the literature, no study has described the precise circumstances during which individuals become aware of an emerging infectious disease prompting Ferguson et al. to call for “*the integrated collation and analysis of epidemiological and, preferably, quantitative social data from epidemics*” so as to make models of lethal epidemics more accurate^5^. Studies on the available information available to citizens during early phases of outbreak are limited. In COVID-19, we found only one study describing the rise of information in China from January 19th to January 26th 2020: this study that people self-limited their mobility before any restrictions, after the release of an official declaration of human-to-human transmission on January 20th, 2020^9^. Another study using 210 answers to an online survey showed that social distancing was not linked to governmental messages: it was performed when individuals perceived a personal health threat^10^.

In this study, we (1) captured both quantitative and qualitative data on the time and circumstances during which academic researchers realized that the COVID-19 pandemic could represent an important event in their lives and for society and (2) compared these results with the evolution of the pre-eminence of COVID-19 in general media, in France between December 2019 and March 2020.

## Results

Our study involved two complementary parts. First, we surveyed academic researchers’ perception on the time and circumstances during which they realized that the COVID-19 pandemic could represent an important event in their lives and for society. We focused on this population because academics are often key in counselling decision-makers in face of unpredictable events with severe consequences and likely to be more susceptible to international events. Second, we appraised of the evolution of the pre-eminence of COVID-19 in general media, in France.

### Online survey investigating when and how did academic researchers become aware of COVID-19 as an important event in their lives and for the society

From March 5th to June 20th 2022, 1568 French academic researchers opened the survey and 868 (55.3%) participated [454 (52.3%) female, median (interquartile range) age: 35 (26–49)]. Among participants, 293 (36.1%) were tenured researchers (including 57 (7.0%) heads of laboratories); 236 (29.1%) were doctoral students and 175 (21.6%) were master students in 2020. All academic disciplines were covered, from Literature (n = 23, 2.8%) and Social sciences (n = 214, 26.1%) to Mathematics and Computer Sciences (n = 124, 15.1%) and Astronomy, astrophysics and Earth sciences (n = 66, 8.0%). In addition, 49 participants (6.0%) were healthcare professionals (Table 1).

**Table 1:** Participants’ characteristics (n = 868).

Characteristic	Number
Age (years)—median (interquartile range)	35 (26–49)
Female gender (n = 838)—n (%)	454 (54)
Position (n = 812)—n (%)	
Head of laboratory	57 (7.0)
Tenured researcher	236 (29.1)
Post-doctoral researcher	17 (2.1)
Doctoral student	236 (29.1)
Master student	175 (21.6)
Physician*	7 (0.9)
Other	57 (7.0)
Academic discipline (n = 820)—n (%)	
Mathematics and computer sciences	124 (15.1)
Astronomy, astrophysics and earth sciences	66 (8.0)
Biology and biochemistry	91 (11.1)
Chemistry	46 (5.6)
Engineering and energy science	30 (3.7)
Physics and material sciences	76 (9.3)
Law and political sciences	29 (3.5)
Literature, linguistics and languages	23 (2.8)
Communication and education sciences	35 (4.3)
Economics	10 (1.2)
Social sciences	214 (26.1)
Epidemiology, biostatistics, public health	36 (4.4)
Medical and pharmaceutical sciences	40 (4.9)
Healthcare professional (n = 811)—n (%)	49 (6.0)

*This figure relates to physicians that do not have another position (e.g., who are not head of laboratory, tenured researchers, doctoral students, etc.).

**Figure 1:**
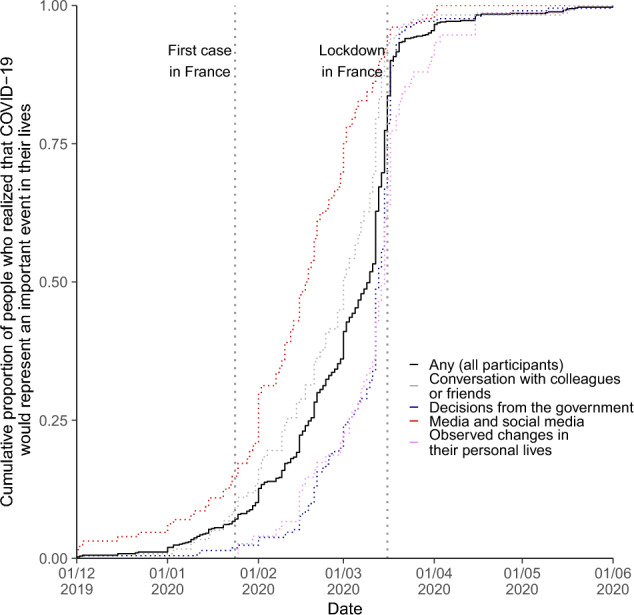
Cumulative proportion of people who realized that COVID -9 was represent an important event in their lives (n = 868).

Among the 845 participants (97.3%) who timed when they realized that COVID-19 was an important event in their lives, 25% did so before February 18th (approx. the date of the first COVID-19 death in France), 50% did so before March 10th (approx. the date of the first presidential allocution about COVID-19 in France) and 75% did so before March 16th (Announcement of the first lockdown in France), respectively (Fig. 1). To put our results into context, before February 2020, the government’s communication was focused on reassuring the population about the low probability of virus spreading in France. However, following the rapid spread of the virus in France from February to March 2020, the government acted several measures against COVID-19: (1) travel restrictions of individuals going and coming from China and from Italy (starting from February 1st 2020); (2) banning of large public meetings on March 9th 2020; and (3) national lockdown, announced on March 16th 2020 and effective on March 17th 2020.

*“[…] The major outbreak in Italy made it certain that COVID would create a global pandemic with extreme disruption to world economies and the need for local closures of one kind or another. In early March, we [me and my family] decided to move to our remote chalet in the Alps. On March 10th, I announced my students that I would be experimenting distance learning over the next few weeks.”* (69 year-old male head of laboratory in Mathematics)

*“At first, I didn’t take the epidemic seriously, despite concerns from my loved ones were concerned. I realized that the pandemic was going to have an important impact on my life when the lockdown was announced in France”* (26 year-old male student in Chemical Engineering)

Circumstances during which participants realized that COVID-19 would disrupt their individual lives were captured using a broad open-ended question “*Do you remember the circumstances (discussion with others, information received, particular event, etc.) during which you realized that the COVID-19 epidemic was an important event in your life?*”. Answers represented a corpus of 185 000 words. Thematic analysis by HB, ERB, NV and VTT led to the identification of nine overarching categories of circumstances during which individuals became aware of COVID-19 as an important event in their lives (Table 2).

**Table 2:** Circumstances during which participants became aware of COVID-19 as an important event in their lives (n = 845).

Circumstances	Verbatim example	Number n (%)
Decisions taken by the government	*“I was coming back to Paris after a week in England. When I saw governmental posters about shielding measures, I realized that "it was really serious". Prevention was becoming systematic and had taken national dimension. The posters looked unusually totalitarian, and for good reason, it was all new for everyone and at the same time it was necessary to act.”* (Female master’s student in History)	302 (35.7)
Information from general or social media	*“At the beginning of 2020, […] I spent nights reading “first hand” news from US and Asian websites, sensing that something worrying was happening […]. Just after New Year's Eve, around January 3rd, I remember I couldn’t sleep at night and I searched for as many international sources as I could.”* (38-year-old male PhD student in social sciences)	209 (24.7)
Conversation with colleagues or friends	*“I heard about {COVID-19] from a friend, in early January 2020. This friend had heard stories of an epidemic in China from her clients who belonged to high public functions. At that time, I was expecting a vaccination campaign like what we had seen for the bird flu. I realized the magnitude of the problem when the epidemic was reclassified as a pandemic.”* (25-year-old female PhD student in mathematics)	189 (22.4)
Observed changes in their personal lives	*“We were waiting for friends at the restaurant. The owner told us that he was going to have to close p for an indefinite period by order of the Prime Minister. The streets seemed strangely empty to us and the city was silent. For me, awareness was long and gradual (…), but the last step taken which led to the acceptance was brutal and took place around the lockdown”* (38-year-old male PhD student in biology)	118 (14.0)
Decisions taken by employer and/or university	*“I was doing fieldwork for my Masters’ internship when I got a call from my supervisor telling me to go home. The fieldwork was getting cancelled because of COVID. That’s when I understood that things were serious”* (25-year-old female master’s student in biology)	78 (9.2)
Observed changes at work	*“I was doing an internship in Colombia, so I was reading information from afar. At first, I didn’t think that it was going to affect me, but when an international seminar was cancelled, I started to feel that it was getting serious. Finally, on March 13 I learned that I had to return to my home country (Spain), because all flights after that date would be cancelled.”* (25-year-old female master’s student in social sciences)	84 (9.9)
Suspected case of COVID-19	*“I live in Mulhouse and we were impacted earlier. Almost all the staff of my children’s day care got sick. Then the children got sick. When I called the director, he told me that something strange was going on. Helicopters were flying over our house more and more often, day and night.”* (38-year-old female PhD student in social sciences)	26 (3.1)
Self-expertise / appraisal of scientific reports	*“As a PhD student working on viral modifications, I started to read up on this emerging virus early […]. I became aware that what was happening was going to be important when I started noticing big contradictions between scientific facts and the official communication.”* (28-year-old female PhD student in biology)	24 (2.8)
Fear for oneself or their entourage	*“At the retirement party of a colleague, a colleague whom I used to greet with a kiss refused to do so. That’s when I realized that the epidemic was scaring people […] and that it could change a lot of things in my relationships with others.”* (40-year-old female researcher in Earth sciences)	24 (2.8)

Three were often associated with an earlier awareness of the disease: (1) information delivered by media or social media (elicited by 24.7% participants); (2) conversation with colleagues or friends (22.4%); (3) self-expertise and/or appraisal of scientific reports (2.8%) (Fig. 1). For example, participants who reported having realized the importance of the COVID-19 pandemic from media and social media were, on average, aware of the disease about 1 month earlier than those who realized this from observed changes in their personal lives. Furthermore, the 21 (2.5%) participants who reported becoming aware of the pandemic from personal contacts with people from China or Italy were often aware of COVID-19 earlier than other respondents (50% before February 24th, and 75% before March 1st).

*“I was on vacation in Singapore in February 2020. I became aware of the magnitude of the pandemic that was coming in view of the precautions taken from Singaporean authorities (disinfection of the subways, taking temperatures in hotels, airports, museums, closing the borders to flights from China from the beginning of February...)”* (60 year-old female researcher in Biology)

The six others were often associated with later awareness of the disease: (1) decisions taken by the government (35.7%); (2) decisions taken by employer and/or university (9.2%); (3) observed changes in their personal lives (14.0%); (4) observed changes at work (9.9%); (5) suspected case of COVID-19 (personal or in their entourage) (3.1%); and (6) fear for oneself or their entourage (2.8%).

*[I realized that COVID-19 was an important event…] "When a scientific event I was attending was cancelled to avoid large gatherings. It was the first time Covid had a direct impact on my life. It was all the more unexpected as the event, planned over 2 days, had taken place the day before but was suddenly cancelled during the second day. Before that, Covid was only a hearsay for me.”* (25 year old female master’s student in Social sciences)

*“I remember that, on February 15th, during a concert of Italian polyphonies, one of the singers stood away because she had been in Milan earlier and thought she had Covid 19. A few days later (less than a week), lockdown was announced in Italy.* (71 year-old male researcher in Biology)

Many participants also reported that awareness came gradually, in several steps. Often, these participants reported the lockdown as the time when they realized the importance of the epidemic despite being “aware” earlier.

*“The impression that Covid was becoming an important event in our lives came gradually. But a turning point was marked in about a week when the internships abroad were cancelled by the university, the confinement in Italy and the wave of deaths in Bergamo.”* (25 year-old male student in Chemistry)

Furthermore, participants’ answers highlight that awareness of the disease did not automatically result in actions. In fact, only few participants reported enacting changes in their activities by themselves.

*“My wife is Italian and my in-laws live in Italy. I could see the epidemic growing there and the denial here. Two weeks before the lockdown, I started moving around exclusively by bike.”* (38 year-old male doctoral student in Social sciences)

Finally, when asked whether participants also anticipated the impact of COVID-19 on society, most participants (n = 626, 72.1%) had not imagined the impact it could have had on society.

“*I was in disbelief. I thought it would be just “a bad and slightly more virulent flu”.”* (41 year-old male researcher in Physics)

Of note, while some participants anticipated the impact on society, they did not realize the importance of COVID-19 in their lives until public health interventions were imposed by governing authorities.

*“"I realized that the COVID-19 outbreak was going to have an impact on my personal life on March 15, when there were rumors about a potential lockdown. Until then, I felt that COVID was an important event in world history, but the outbreak had not yet impacted my life."»* (59 year-old male researcher in Computer Sciences)

### Media appraisal investigating the evolution of the pre-eminence of COVID-19 in comparison to other topics, over time in a newspaper, a television and a radio show

We compared the time when academic researchers became aware of COVID-19 as an important event in their lives and the evolution of the pre-eminence of COVID-19 in the general media, in France.

A single investigator (HB) appraised the evolution of the pre-eminence of COVID-19 in comparison to other topics, over time, in three general media sources: (1) the daily national newspaper “Le Monde ”, which is one of the French newspapers of record; (2) the evening news “Journal de 20 h” from “France 2”, which is one of the main public French television (TV) show; and 3) the morning news “*Journal de 8 h*” from “*France Inter*”, which is one of the public radio news show with the largest audience in France.

**Figure 2:**
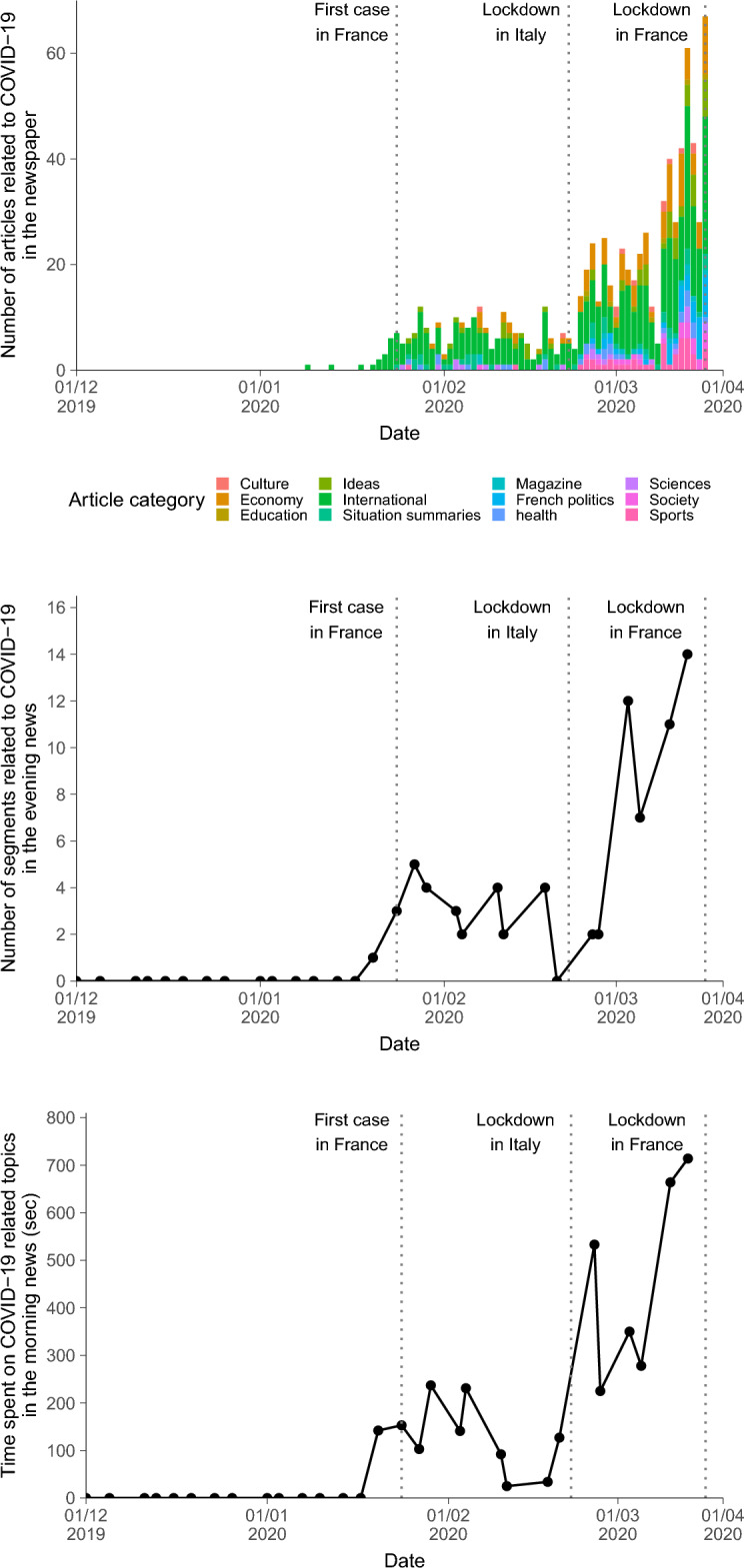
Media appraisal of the evolution of the preeminence of COVID-19 in a national newspaper (**A**), television evening news (**B**) and radio morning news (**C**).

In total, we extracted 820 articles from “*Le Monde*” related to COVID-19. Two articles were published between the 1st of January and the 15th of January whereas 67 articles were published in one day, on March 16th. Besides the increase in the number of articles related to COVID-19, we observed a spill over in all areas of society from Economy to Sports (e.g., cancellation of cultural or sport events). In Fig. 2A, February 22nd, just after the lockdown in Italy, marks the timepoint after which almost all sections of the newspaper are related to COVID-19.

In the evening news, we appraised 30 broadcasts selected at random from December 2, 2019 to March 17, 2020. In our sample, the first mention to COVID-19 was about concerns about the evolution of COVID-19 during the Chinese New Year (January 20th). Similar to the newspaper, Fig. 2B we observed a rapid increase in the number of mentions to COVID-19 starting from the end of February. In general, the evening news involves about 10–15 segments on various topics, meaning that starting from March 1st, almost all the broadcast was dedicated to COVID-19.

In the radio morning news, we appraised 30 broadcasts selected at random from December 2, 2019 to March 17, 2020. In our sample, the first mention of COVID-19 was on January 22nd after an emergency meeting of the World Health Organization. Similar to the other media, Fig. 2C shows a rapid increase in the number of mentions to COVID-19 starting from the end of February. After March 12th and 13th, over half of the morning news time was dedicated to COVID-19.

Overall, we observed a similar trend in all three media, COVID-19 was (1) rarely mentioned prior to January 2020; (2) regularly mentioned from January 15th to March 1st with about 20% of the topics and time of TV and radio shows dedicated to the subject; and (3) became the most preeminent topic discussed in all three media, with a spill over in all areas of society after March 1st.

## Discussion

In this mixed methods study, we appraised the time and circumstances during which academic researchers realized that COVID-19 was an important event in their lives and society. In particular, we showed that awareness came from nine categories of circumstances: (1) decisions taken by the government; (2) information from media or social media; (3) conversation with friends; (4) observed changes in their personal lives; (5) decisions taken by the employer; (6) observed changes at work; (7) suspected case of COVID-19 in their entourage; (8) fear for oneself or their entourage and (9) self-appraisal of scientific reports.

During the analysis of qualitative data from this study, we also found that awareness of an epidemic is a progressive process with three stages: first participants “heard” of COVID-19 and/or followed news from China without believing that the epidemic could become their reality. Exposition to information was “passive” at that stage. Second, participants realized that the epidemic was closer with the first cases in France and the Italian lockdown. They often became “active” in their search of information and began to worry. Finally, they became aware that COVID-19 was going to be an important event in their lives when the lockdown became “inevitable” in France. We hypothesize that situations encountered gradually transform of our vision of the world: uncertainties take shape and our ways of sensing the what is at stake changes, especially as the impact become tangible in the close environment.

Interestingly, our results show a similar trend in the time when media become “interested” in COVID-19, in France and in China. One study using data from social media in China showed that before January 19th 2020, there was little information online about COVID-19, with an exponential increase in the number posts and comments from January 19th to January 26th 2020^9^. Similar, our data shows that COVID-19 was not mentioned before January 19th in French media, although the epidemic was notable in China at that time. This contrasts with the following massive “infodemic” which made it difficult for scientists and governments to communicate and spread information to citizens. In all, early communication is critical to spread awareness. Afterwards, the cacophony of mis- or event disinformation (“fake news”) may mitigate the efficacy of communication, disease awareness and health behaviours.

Our study has several limitations. Regarding the survey, it is retrospective and may be affected by memory bias. To minimize memory bias, we asked respondents to use agendas, mails and text messages when answering the questionnaire. Yet, in our data, we observe that most participants provided precise dates and circumstances, suggesting that they usually remember a “turning point” when they realized the importance of the pandemic. Second, our population is not representative of academic researchers in France. We tried to mitigate selection bias by opting systematically contacting all doctoral schools, in France. However, only willing researchers participated in the survey. Furthermore, we did not make any reminder invitation mails, to avoid spamming researchers. Despite these limitations, our results show that our sample was diverse, covering all disciplines and seniority levels, from PhD students to head of laboratories. Third, although the questionnaire was developed from qualitative interviews with questions and wording developed so as to provide the most meaningful answer, we did not pilot test it in its final form.

Regarding the media analysis, we selected two were public media and may have similar editorial policies. Second, we chose a limited number of time points, lowering the precision of the evolution curve. Finally, analysis was performed by a single investigator.

To conclude, as some participants reported that the awareness of an emerging epidemic disease led them to take actions in their personal lives or in their laboratory, we may want to consider the process of awareness of an emerging epidemic disease as leading to a “turning point” during which our appraisal of the world changes^11^. As Abbott points out, our appraisal of the world and our social interactions are part of a permanent construction based on multiple factors^12^. A turning point is a process that intervenes in a network of established and ongoing interactions within which emerge new orientations. It is a long-term process and has two key points: possibility and action. In early 2020, it was potentially feasible (the possibility) for a pandemic to affect participants. But it only became real when it effectively transformed their daily lives (the action). The turning point is contingent insofar as the outcome depends on the sequence of these internal events. And it is these events that a cross-methodology, such as the one we proposed, may be able to describe. The heuristic interest of the notion of turning point is therefore based on its situated and procedural character. These first results lead us to wonder about the outcome of this long-term process and to what extent we are still in the process of turning point initiated by the pandemic.

## Conclusion

Academic researchers who reported realizing the importance of COVID-19 from media and social media became, on average, aware of the disease about 1 month earlier than those who realized this from observed changes in their lives (often linked to governmental decisions). In parallel, we show that COVID-19 became a preeminent topic in the French general media only after March 1st 2020. These combined results hint that public institutions willing to accelerate the spread of awareness of an emerging disease abroad, may want to implement a massive media strategy early. Second, our results also show that awareness seems to be directly related to visible changes in peoples’ lives (i.e., cases in their entourage, decisions from their employers, decisions from the government); implying that response to an emerging infectious disease must be rapid and visible, so as to prompt awareness and modify behaviour that may prevent the epidemic.

## Materials and methods

This study involved two complementary parts. First, we surveyed academic researchers’ perception on when they realized that COVID-19 pandemic was an important event in their lives and for society. Second, we appraised of the evolution of the pre-eminence of COVID-19 over other topics in the media. All methods were carried out in accordance with relevant guidelines and regulations.

The study is a social science study and does not require Ethical approval before its conduct (https://www.legifrance.gouv.fr/jorf/id/JORFTEXT000025441587/—French law). As such, it does not require written informed consent from subjects. All participants were informed of the study objectives, modes of data collection and analysis methods before participating and provided electronic consent before filling the questionnaire.

### Online survey investigating when and how did academic researchers become aware of COVID-19 as an important event in their lives and for the society

We surveyed academic researchers’ perception on the time and circumstances during which they realized that the COVID-19 pandemic could represent an important event in their lives and for society. We focused on this population because academics are often key in counselling decision-makers in face of unpredictable events with severe consequences.

Participants were academic researchers (master, PhD students, postdoctoral fellows and tenured researchers), in France, recruited by a mail invitation sent to the 275 doctoral schools in France^13^. They were invited to connect on a secured website to answer a single online questionnaire, in March 2022. We chose to focus on this population because they are often key in counselling decision-makers in face of unpredictable with severe consequences. Participants who had been invited were encouraged to invite relatives and friends who were eligible to participate, by a ‘snowball’ sampling method which involves identifying an initial number of participants who serve as ‘seeds’ to help identify peers who, in turn, are asked to invite others and so forth^14^.

The questionnaire was designed from 10 face-to-face interviews with participants following methods recommended to develop participant reported measurement tools^15^. This approach helped us understand which questions and words prompted the most meaningful answers from participants. Furthermore, the open-ended interviews helped in the definition of the constructs to be addressed—particularly, around the concept of “turning point”. The questionnaire started with a graphical representation of major events that occurred from December 2019 to March 2020 (e.g., first case of COVID-19 in France, dates of presidential allocutions, etc.) to help respondents remember isolated events by making use of the interrelatedness of autobiographical experiences and to remobilize distant memory of facts which took place several months apart^16,17^.

Then, respondents were asked to describe the circumstances during which they realized that the COVID-19 pandemic was an important event in their lives and for society, using a broad open-ended question “*Do you remember the circumstances (discussion with others, information received, particular event, etc.) during which you realized that the COVID-19 epidemic was an important event in your life?*”. Finally, respondents were asked to date this event. They were encouraged to use agendas, mails or messages from this period, to provide accurate dates.

Sample size was determined by our two objectives. First, we aimed at determining the time when academic researchers became aware of COVID-19 as an important event in their lives and for the society. As this objective was descriptive, a larger sample size would result in more precision. For that, we adopted a pragmatic approach and used the maximal sample feasible^18^. Second, the survey aimed at identifying the circumstances during which academic researchers became aware of COVID-19 using a qualitative approach. Simulation studies have shown that 150 participants and over are adequate for online surveys with open ended questions^19^.

Open-ended answers were analysed by four researchers (HB, EBR, NV and VTT) using thematic analysis. The analysis aimed at understanding the circumstances during which the Covid-19 pandemic became an “event” identified as such in a large population of academic researchers; it was therefore structured by constructs that could capture the transition between what happened “before” the pandemic and the emerging awareness that researchers were “in” the pandemic. First, the content collected through the semi-structured interviews was reviewed and refined to identify recurrent patterns of perceived “turning points”—constructed retrospectively by the researchers’ accounts of the way they experienced the early days of the pandemic. Second, we used this knowledge to analyse the survey data in three steps. First, during an initial reading and coding of the 100 first respondents’ answers, one researcher (HB) inductively coded the manifest content of the first 100 answers and identified “in vivo codes”: literal terms used by participants to describe the circumstances during which they realized the pandemic was an important event in their lives. Second, the researchers developed a list of categories by grouping the previously defined codes by similarity. Finally, one investigator (HB) assigned all remaining answers to the categories defined earlier. Whenever a new idea emerged, researchers discussed the idea, thereby refining and enriching the list of categories.

We presented the cumulative number of participants who realized that the COVID-19 pandemic represented an important event in their lives, over time. Dates before December 2019 and after December 2021 were doubled checked using the open-ended answers to account for errors in years (e.g., often participants dated their awareness in 2021 while referring to the first lockdown in France). Whenever the open-ended text could not be used to appraise whether there was an entry error (n = 23), we considered the date as missing information.

### Media appraisal investigating the evolution of the pre-eminence of COVID-19 in comparison to other topics, over time in a newspaper, a television and a radio show

A single investigator (HB) appraised the evolution of the pre-eminence of COVID-19 in comparison to other topics, over time, in three general media sources: (1) the daily national newspaper “*Le Monde*”, which is one of the French newspapers of record; (2) the evening news “*Journal de 20 h*” from “*France 2*”, which is one of the main public French television news show; and (3) the morning news “*Journal de 8 h*” from “*France Inter*”, which is one of the public radio news show with the largest audience in France.

In the daily national newspaper “*Le Monde*”, the investigator searched the archives (available online at http://www.lemonde.fr/archives-du-monde) between the December 2, 2019 and March 17, 2020 for all articles that included the terms “coronavirus”, “-ncov”, “covid” or “virus” in the title or summary. Entries retrieved were screened to exclude irrelevant articles, for example on Ebola, Papilloma or Computer viruses. For each article we extracted its publication date, title, and summary as exhibited on the article’s webpage.

For the evening news “*Journal de 20 h*” from “*France 2*”, the investigator selected two random broadcasts every week, between the December 2, 2019 and March 17, 2020 (available online at http://www.francetvinfo.fr/replay-jt/france-2/20-heures). The investigator measured the time dedicated to any COVID-19 topic (in minutes) during the broadcast. In addition, he counted, for each broadcast, the number of topics related to COVID-19.

For the radio program “*Journal de 8 h*” from “*France Inter*”, the investigator selected two random broadcasts every week between December 2, 2019 and March 13, 2020 (available online at http://www.radiofrance.fr/franceinter/podcasts/le-journal-de-8h). The investigator measured the time dedicated to any COVID-19 topic (in minutes) during the broadcast. In addition, he counted, for each broadcast, the number of topics related to COVID-19.

## Data Availability

The datasets used and/or analyzed during the current study available from the corresponding author on reasonable request.
